# Next-generation universal hereditary cancer screening: implementation of an automated hereditary cancer screening program for patients with advanced cancer undergoing tumor sequencing in a large HMO

**DOI:** 10.1007/s10689-022-00317-w

**Published:** 2022-10-20

**Authors:** Trevor L. Hoffman, Hilary Kershberg, John Goff, Kimberly J. Holmquist, Reina Haque, Monica Alvarado

**Affiliations:** 1 Department of Regional Genetics, Southern California Permanente Medical Group, Pasadena, CA USA; 2 Department of Research & Evaluation, Southern California Permanente Medical Group, Pasadena, CA USA; 3 Dept. of Health Systems Science, Kaiser Permanente Bernard J. Tyson School of Medicine, 91101 Pasadena, CA USA

## Abstract

Variants in hereditary cancer risk genes are frequently identified following tumor-based DNA sequencing and represent an opportunity to diagnose hereditary cancer. We implemented an automated hereditary cancer screening program in a large HMO for all patients who underwent tumor-based DNA sequencing to identify patients with hereditary cancer and determine if this approach augmented existing genetic counseling approaches driven by personal/family history criteria. Regular automated searches of a centralized tumor DNA variant database were performed for ATM, BRCA1, BRCA2, MLH1, MSH2, MSH6, PALB2, and/or PMS2 variants, and germline hereditary cancer gene panel testing was offered to patients with tumor variants who had never undergone germline testing. Patients completing germline testing due to their tumor DNA test results were considered part of the tumor DNA safety net. Patients previously completing germline testing via traditional genetic counseling and tumor DNA safety net were compared for demographics, tumor type, presence of germline pathogenic/likely pathogenic (P/LP) variant, and whether NCCN criteria were met for hereditary cancer genetic testing. Germline P/LP variants were common in both groups. Patients who received germline testing through traditional genetic counseling were more likely to have cardinal hereditary tumors than the tumor DNA safety net group. Patients identified with hereditary cancer through traditional genetic counseling were more likely to meet NCCN personal/family history criteria for germline testing than the tumor DNA safety net group (99% versus 34%). A universal tumor DNA safety net screen is an important diagnostic strategy which augments traditional genetic counseling approaches based on personal/family history.

**Supplementary Information:**

The online version contains supplementary material available at 10.1007/s10689-022-00317-w.

## Introduction

Identifying patients with hereditary cancer syndromes has relied on multiple complementary strategies. Individuals with a personal and/or family history of certain cancers have typically been offered hereditary cancer genetic testing using various criteria that have evolved as the number of known hereditary cancer syndromes has grown and testing access has increased. These approaches rely on recognition of characteristic personal and/or family history elements which are not always present due to the incomplete penetrance and variable expressivity for various high/moderate risk hereditary cancer syndromes. Once a patient has been diagnosed with hereditary cancer through germline DNA testing, cascade testing of family members is an important method for diagnosing additional cases or identifying those at high risk.

Another important tool for diagnosing hereditary cancer has been universal tumor screening. The application of MMR-immunohistochemistry screening to colon and uterine cancer specimens has been a cost-effective and effective strategy to diagnose individuals with Lynch syndrome [1]. Universal screening approaches such as MMR-immunohistochemistry have the distinct advantage over criteria-based testing in that they do not rely on personal and family history information, which are frequently not present or go unrecognized by providers. However, universal MMR-immunohistochemistry is somewhat limited in that it only applies to a single hereditary cancer predisposition syndrome (Lynch syndrome) among the dozens of hereditary cancer conditions which are now routinely diagnosed in the clinical setting.

As the expansion of tumor-based DNA sequencing and precision medicine approaches have revolutionized cancer treatment, an important new opportunity to diagnose hereditary cancer has arisen. Tumor variants in many hereditary cancer predisposition genes act as tumor-suppressors, drive cancer progression, and can guide novel cancer therapeutics [2]. Tumor DNA testing is being routinely used for optimal drug selection, and evidence is emerging that variants in multiple different hereditary cancer genes identified through tumor DNA sequencing are also frequently present in the germline [3, 4]. Our goal was to study the application of a universal tumor screening strategy based on tumor DNA test results as a method to diagnose hereditary breast/ovarian cancer and Lynch syndrome in Kaiser Permanente Southern California, a large HMO with 4.7 million members with a diverse ethnic background. We found that a universal tumor DNA variant screening strategy was an effective method to diagnose patients with these moderate/high risk hereditary cancer conditions. Furthermore, patients diagnosed with hereditary cancer through a universal tumor DNA strategy often had atypical cancer types for their hereditary cancer syndrome, and they frequently did not meet current personal/family history guidelines for hereditary cancer genetic testing. Thus, a universal tumor DNA testing screen for the diagnosis of hereditary cancer represents a novel and important adjunctive diagnostic approach to personal/family history-based methods that should be more broadly applied within healthcare delivery systems.

## Methods

### Subjects and study setting

Study participants were drawn from Kaiser Permanente Southern California (KPSC), an integrated non-profit health care delivery system with over 4.7 million ethnically diverse members, 15 medical centers, and a network of 6,200 physicians. Since 2014, KPSC implemented inherited cancer susceptibility testing via a hereditary cancer multigene panel for selected patients with clinical presentations or family histories that suggested a hereditary cancer syndrome. The genetic counselors and geneticists at the KPSC healthcare system follow guidelines of the U.S. National Comprehensive Cancer Network (NCCN) for referrals for genetic counseling and testing for hereditary breast, ovarian, pancreatic and colorectal cancers [5, 6]. Clinicians at each of the 15 KPSC medical centers throughout southern California can refer their patients to a genetic counselor. Additionally, because KPSC is an integrated healthcare system, genetics providers can contact selected patients directly to offer genetic testing and counseling. All study design, data acquisition, and data analysis was performed in accordance with approval and oversight by the KPSC Institutional Review Board.

### Data elements

Beginning in May 2019, patients in KPSC with advanced stage III/IV cancers due to a solid tumor that was considered treatment refractory by their treating oncologist could be offered tumor DNA sequencing (Strata NGS, Strata Oncology) for clinical management purposes [7], where the tumor DNA test results were collected and stored in a centralized database. Genes analyzed by tumor DNA sequencing included full-gene coding exons including 5 base pairs of flanking intronic sequences along with copy number variant analysis. Variants reported had allele frequencies > 5%. Tumor variants reported included: frameshift, nonsense, splice-site alterations impacting the +(-)1 or +(-)2 position, missense variants with at least one ClinVar LP/P interpretation, and intra/whole gene deletions. The StrataNGS test is validated to detect and report insertions/deletions (indels) up to 15 bases in length, though larger indels were also reported if detected (though sensitivity is reduced for indels > 15 base pairs). StrataNGS tumor variant reporting did not specifically include an interpretation of variant pathogenicity; however, the reporting strategy is strongly skewed towards LP/P variants but likely includes some (missense) VUS. Starting in 9/2020, we searched a tumor DNA database for all tumor samples with a reported tumor variant in the following gene list: ATM, BRCA1, BRCA2, MLH1, MSH2, MSH6, PALB2 and PMS2. We continued to perform regular screening of patient tumor samples from the tumor DNA database for the above gene list monthly as new cases were added from 10/2020 to 04/2021 when the study group was closed (Supplemental Fig. 1). Any patient from 5/2019 to 4/2021 identified with a tumor variant in the gene list of interest was included. For the very small number of patients having a tumor variant in this gene list who underwent ≥ 2 tumor DNA tests, the first chronological tumor DNA test was used for data analysis. Any subsequent samples were excluded; thus, each tumor sample analyzed represented a single unique patient. In all cases where an individual had multiple biopsy samples sent for tumor DNA sequencing, every tumor would have met the inclusion criteria. Information about the patient’s primary cancer diagnosis was abstracted from manual electronic medical record chart review. In some cases, a primary tumor site was unknown due to advanced disease stage at the time of cancer diagnosis and was labelled as “unknown primary”. Final determination of variant pathogenicity was determined based on the commercial laboratory’s interpretation of germline test, which is based on industry-standard published guidelines [8]. Patient age was set at the chronological age at the date of the tumor biopsy results. Race/Ethnicity data was abstracted from the KPSC electronic medical record system.

### Identification of patients for the tumor DNA safety net

Each patient with a tumor DNA variant in the gene list of interest was evaluated for prior germline hereditary cancer genetic testing via a germline hereditary cancer genetic testing database. Any patient with a tumor DNA variant in the gene list of interest who had not previously undergone hereditary cancer testing and was still an active KPSC member was contacted and offered genetic counseling and germline hereditary cancer panel testing by a genetic counselor or medical geneticist. The automated portion of the screen involved computer-based searches of the tumor database by a computer analyst who determined which patients had not undergone previous germline testing and provided patient lists to genetics staff members for outreach. All genetic counseling visits that were completed as a result of the tumor DNA screening process occurred between 09/2020 and 08/2021. Germline hereditary cancer genetic tests (either before or after tumor sequencing was completed) were performed via NextGen sequencing in CLIA-certified commercial laboratories (GeneDx, Invitae Genetics, or Myriad Laboratory) for a multi-gene hereditary cancer gene panel including all genes of interest using blood, buccal or saliva sampling. Genetic counseling visits included collection of a 3-generation pedigree and a clinical determination of whether the patient met current NCCN guidelines for hereditary cancer genetic testing [5, 6]. All patients who underwent hereditary cancer genetic testing prior to tumor DNA testing were evaluated considering NCCN guidelines for germline testing at the time of their test which are used to determine eligibility for testing in the KPSC health plan. Any patient without prior germline genetic testing who was identified as a result of having a tumor variant in the gene list of interest was offered germline testing whether or not they met NCCN guidelines for hereditary cancer genetic testing. All patients who underwent hereditary cancer genetic testing as a result of the tumor DNA screening process were provided post-test genetic counseling about their test results.

Patients were considered part of the traditional genetic counseling model group if: (1) their germline DNA test occurred prior to the tumor DNA testing, or (2) if they had germline testing on a date after their tumor DNA test for reasons that were unrelated to the tumor DNA test results (determined through manual chart review to assess the reason for referral to the genetics department for testing and any additional documentation regarding tumor DNA test results). Patients were considered part of the tumor DNA safety net if: (1) they were referred to genetics by oncology providers because of their tumor DNA test results (as stated in the oncologist’s indication for referral to genetics), or (2) if they were contacted directly by genetics providers and offered germline testing based on tumor DNA test results due to regular interval searches of the tumor DNA variant database.

### Statistical analysis

Given the descriptive evaluation of this safety net program and small sample size (503 tumor specimens from patients), we examined the percentages of tumor variants found to be germline by genes of interest as well as numbers germline variants in different primary tumors [9]. Differences in the percent of patients with hereditary cancer meeting NCCN Guidelines in the traditional genetic counseling group versus the tumor DNA safety net group was examined using Fisher’s Exact test.

## Results

### Patient groups and demographics

**Fig. 1:**
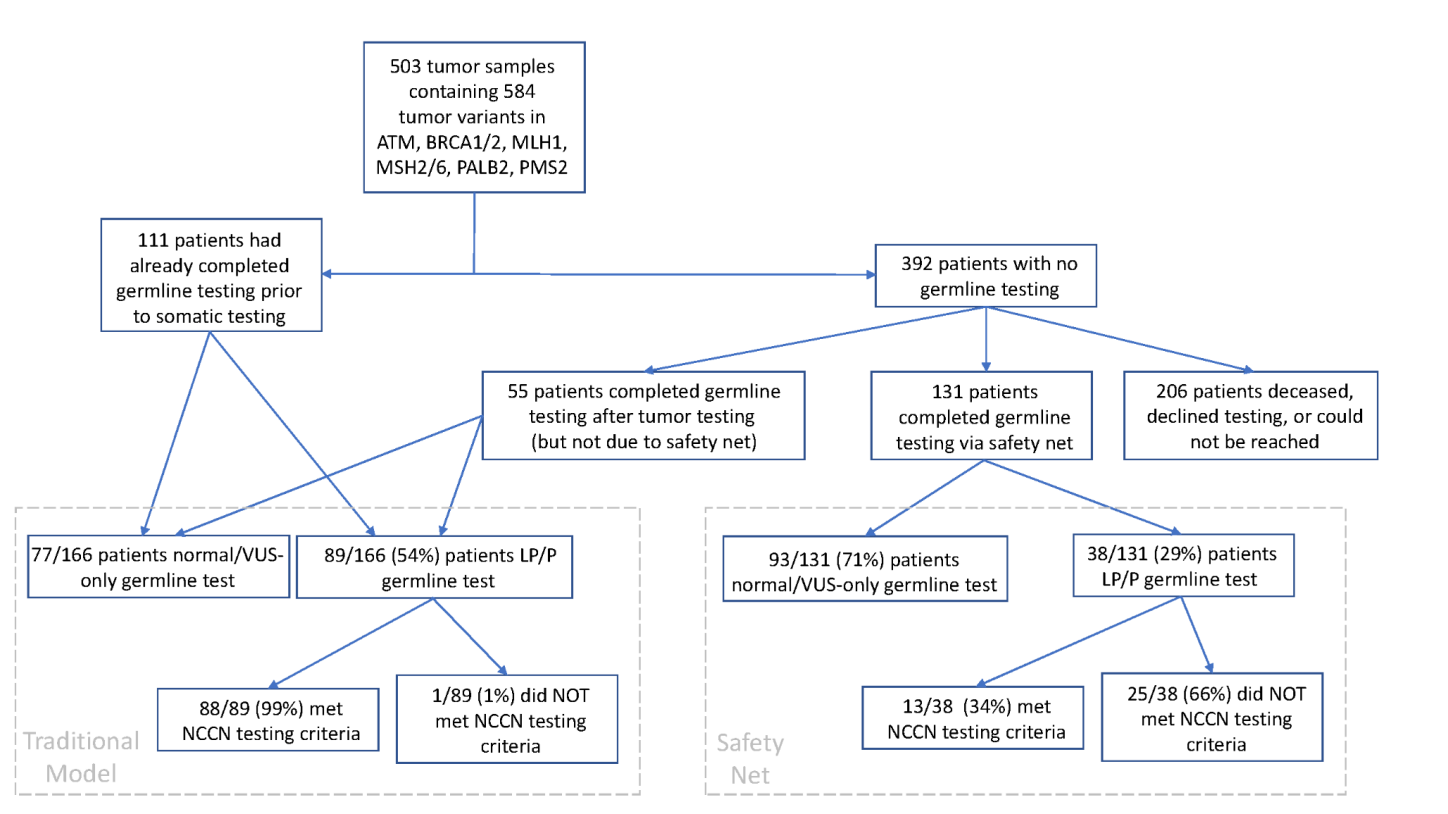
Identification of tumor DNA safety net patients. A total of 503 tumors were identified with tumor variants in at least one of the following genes: ATM, BRCA1, BRCA2, MLH1, MSH2, MSH6, PALB2, PMS2. Patients were grouped into the traditional genetic counseling group who: (1) underwent germline testing prior to tumor DNA testing, or (2) underwent germline testing after tumor DNA testing for personal/family history indications and not due to their tumor DNA results as verified by chart review. Patients who were contacted and completed germline testing due to a tumor DNA result constituted the tumor DNA safety net group. For patients who had a likely pathogenic/ pathogenic germline variant in the gene list of interest, a genetic counselor determined whether they met NCCN guidelines for germline testing based on personal/family history

From a total of 6830 tumor samples from 6527 individual patients that underwent tumor DNA sequencing, 584 unique tumor variants were identified in the gene list of interest (ATM, BRCA1, BRCA2, MLH1, MSH1, MSH6, PALB2 and PMS2) from 503 unique tumor samples/patients during the study period (Fig. 1). 111 patients had previously undergone germline testing prior to completing their tumor DNA test and were considered part of the traditional genetic counseling group (where a patient was referred for genetic counseling and germline testing due to personal/family history criteria and/or cascade testing). Among the 392 patients who had not undergone germline testing at the time of their tumor DNA testing: (1) 52.6% (206/392) patients never completed germline testing (they were deceased, declined germline testing or could not be contacted), (2) 14.0% (55/392) patients completed germline testing concurrently/after their tumor DNA test for a personal/family history indication unrelated to the tumor DNA testing results (this group also comprised part of the traditional genetic counseling group), and (3) 131/392 (33.4%) patients completed germline testing specifically due to their tumor DNA result or searches of the tumor variant database resulting in direct outreach by the genetics department and germline testing.

Age and race/ethnic distribution for the different patient groups is shown in Table 1. The average age for the traditional genetic counseling group, tumor DNA safety net group, and patients that did not complete germline testing was ≥ 60 years (60, 65, and 67 years, respectively). The traditional genetic counseling group was comprised of more female patients (74% female, 26% male) than the tumor DNA safety net group (54% female, 46% male) and the group that did not complete germline testing (43% female, 57% male). There was a diverse ethnic composition in all groups (12% African-American, 14% Asian, 25% Latin American/Caribbean, 45% Northern/Western European, and 5% other/unknown for the total cohort). There were slightly fewer African American (7%) and more Latin American/Caribbean (28%) patients in the traditional genetic counseling model than in the other groups (13–16% and 23–24%, respectively).

**Table 1:** Patient Demographics

	Age Characteristics (yrs)			Biological Sex		Race/Ethnicity				
	Median	Average	Range	Female	Male	Africa	Asia	Latin Am Caribbean	NW Europe	Other/Not Provided
All Study Patients	65	64	23–95	56%	44%	12%	14%	25%	45%	5%
Traditional GC Model	61	60	26–82	74%	26%	7%	16%	28%	44%	5%
Tumor DNA Safety Net	66	65	28–93	54%	46%	13%	13%	24%	46%	4%
Unable to contact/refused/deceased	68	67	23–95	43%	57%	16%	12%	23%	45%	5%

### Germline and tumor DNA test results

In the traditional genetic counseling group that completed germline testing, 54% (89/166) of patients harbored a pathogenic or likely pathogenic (P/LP) germline variant in at least one of the genes of interest, while 46% (77/166) had a germline test that was negative/normal or contained one or more variants of uncertain clinical significance and no LP/P variants (VUS only). In the tumor DNA safety net group that completed germline testing, 29% (38/131) of patients had at least one germline P/LP variant, while 71% (93/131) of patients had a negative or VUS-only result on germline testing. Among patients with a germline P/LP variant, significantly more patients met criteria under NCCN breast/colon guidelines for germline testing in the traditional genetic counseling group (99%; 88/89) compared to the tumor DNA safety net group (34%; 13/38) with p < 0.001 (Fisher’s exact test).

**Fig. 2:**
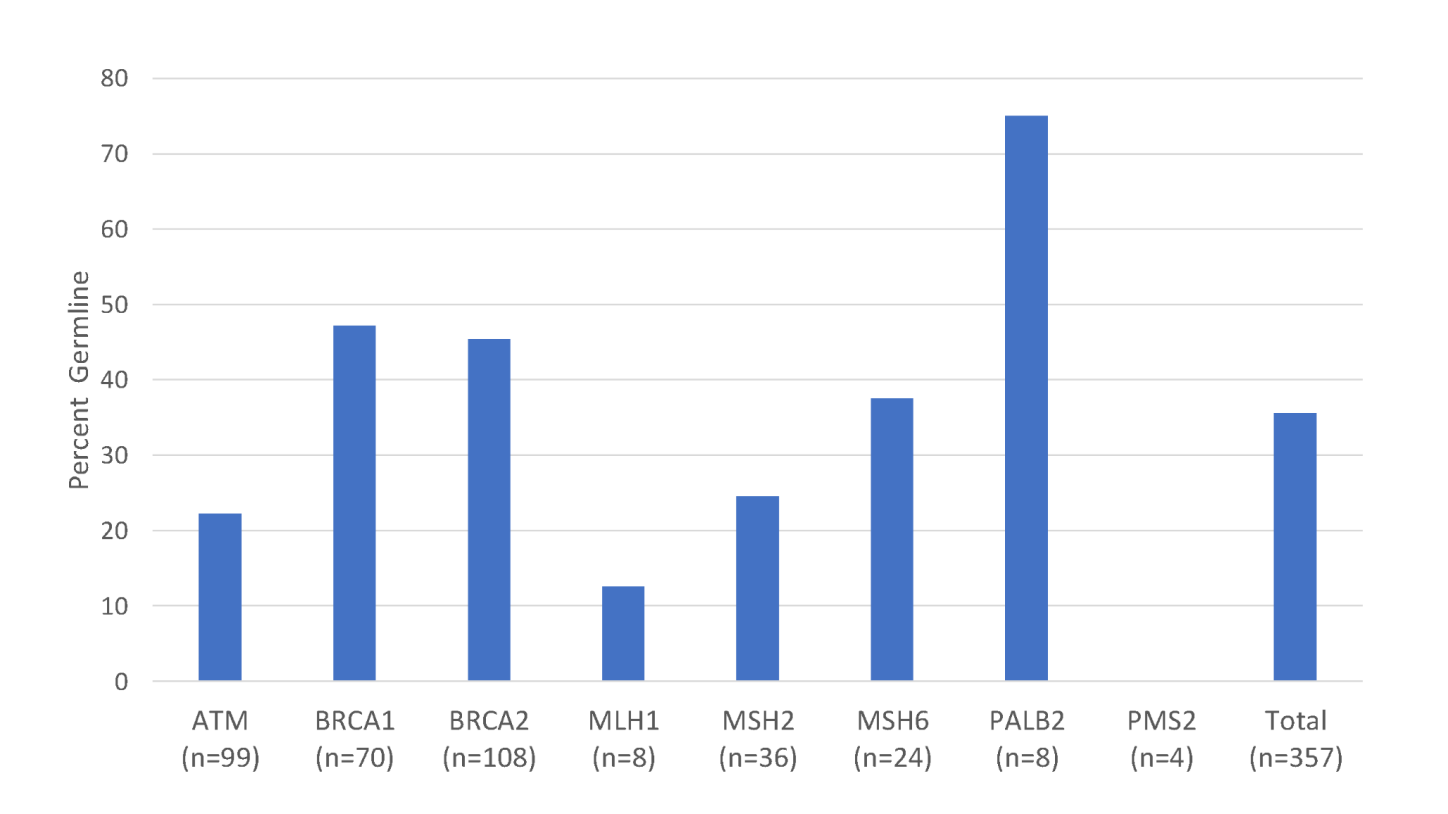
Percent of tumor variants found to be germline. A total of 297 patients were found to have a tumor variant in at least one of the genes of interest (ATM, BRCA1, BRCA2, MLH1, MSH2, MSH6, PALB2) who also underwent germline testing. A total of 357 tumor variants were identified in the gene list of interest. The percentage of patients having a likely pathogenic/pathogenic germline variant in a gene where their tumor had at least one tumor variant in that gene is indicated

For all patients who completed germline testing, we also determined the percentage of P/LP tumor variants that were germline for each of the 8 genes (Fig. 2). For genes where at least 20 tumor variants were identified in the tumor data set (ATM, BRCA1/2, MSH2, MSH6) between 20 and 50% of these tumor variants were also germline. Genes with fewer than 10 tumor variants identified (MLH1, PALB2, PMS2) had more variable frequencies of being germline (likely due to low n value for each of these genes), but collectively they had a similar percentage of being germline when combined as a single group (40%). Across all genes, 36% (127/357) of the P/LP tumor variants identified were present in the patient’s germline. In all cases where a germline P/LP variant was identified, the identical variant was seen in tumor DNA sequencing.

### Analysis of primary tumor types

**Fig. 3:**
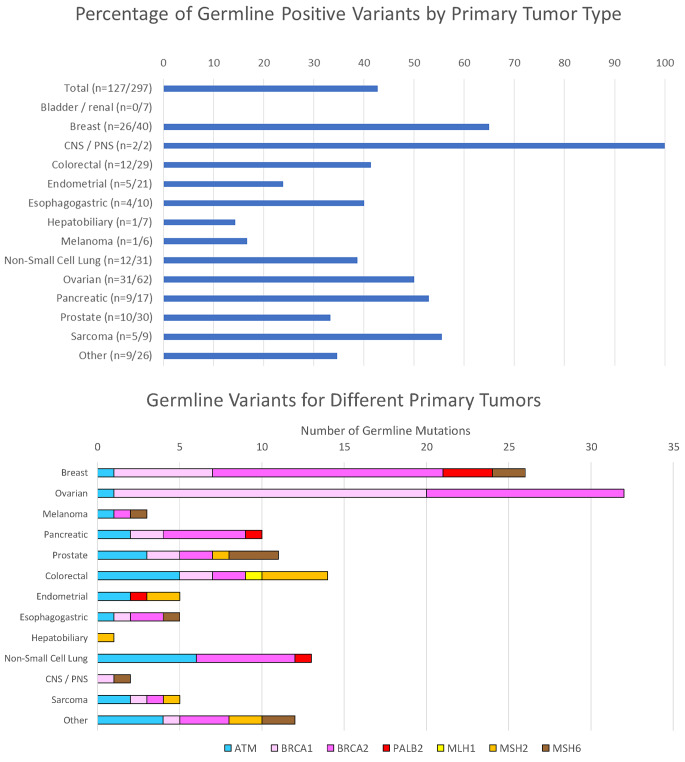
Panel (A) Distribution of germline variants among different tumor types. For tumor samples containing a DNA variant the genes of interest (ATM, BRCA1, BRCA2, MLH1, MSH2, MSH6, PALB2) the primary tumor site and likelihood of finding a likely pathogenic or pathogenic germline variant in one of these genes is shown. The number of likely pathogenic/pathogenic germline and tumor variants is listed for each category in parentheses and the bars indicate the percentage of germline variants. Panel (B) The individual number of germline variants for each specific gene for each of the different primary tumor types is shown.

We also determined the primary tumor type for all patients who underwent germline testing and the likelihood for a germline variant to be present in different primary tumors with a tumor variant (Fig. 3). Collectively, 43% (127/297) of all tumors with a variant in the gene list of interest also harbored a germline variant. The most frequent primary tumors containing a tumor variant (in decreasing order) were breast, pancreatic, ovarian, colorectal, lung, and prostate (Fig. 3a). Other tumor types (e.g. melanoma, bladder, hepatobiliary, sarcoma) represented a smaller proportion of the overall tumor group, but these tumors contained a collectively similar frequency of germline variants to more common tumors analyzed. The distribution of germline variants found among different primary tumors is shown in Fig. 3b. Germline variants in BRCA1/2 were represented at high frequencies among breast, ovarian, prostate and pancreatic tumors, although germline BRCA1/2 variants were also found in primary lung, sarcoma, and other tumor types (at a lower proportion of the total). Variants in the Lynch syndrome genes (MLH1, MSH2, MSH6, and PMS2) were seen more commonly in colorectal and prostate cancer than other cancer types but were not limited to these tumors. Germline variants in the ATM gene were found more consistently across multiple primary tumor types and demonstrated a more consistent fraction of the overall germline variant burden than hereditary breast cancer (BRCA1/2) or Lynch syndrome genes in different tumors. There were only a small number of tumors with variants in PALB2 (n = 8; 7 of these variants were germline).

**Fig. 4:**
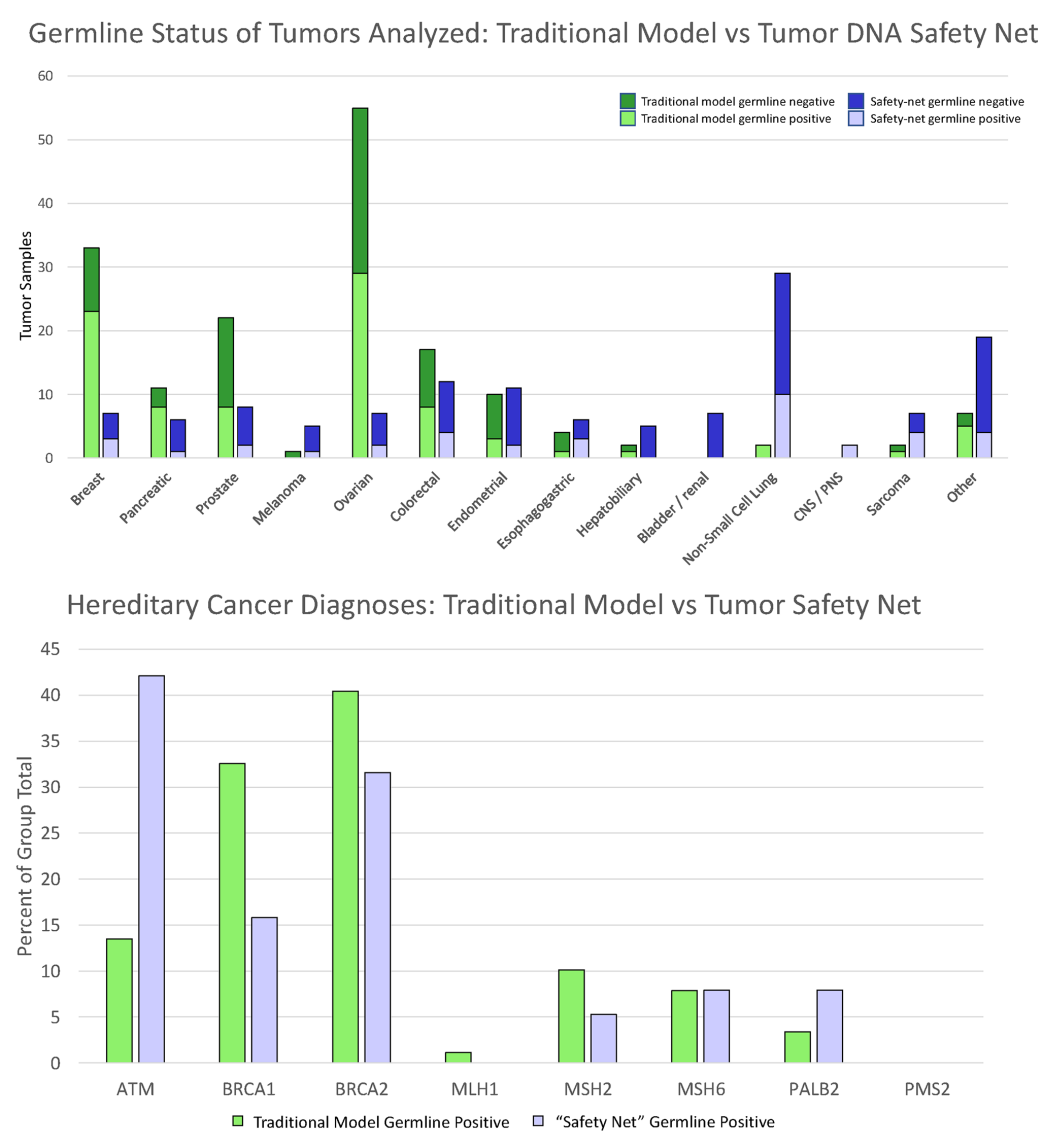
Distribution of primary tumor types, germline variant status, and distribution of germline variants found in traditional genetic counseling versus tumor DNA safety-net patients. Panel (A) The number of patients with different primary tumor types are shown for patients identified through the traditional genetic counseling (left bar for each tumor type) and the tumor DNA safety net (right bar for each tumor type). Patients with germline variants are shown in the lighter shade, while patients who only had a somatic (non-germline) tumor variant are shown in the darker shade. Panel (B) The distribution of the different germline hereditary cancer gene variants are shown for the traditional genetic counseling (left bar for each gene) versus tumor DNA safety net (right bar for each gene) as a percentage of each group

We also determined the distribution of primary tumor types and distribution of germline variants found in the traditional genetic counseling model versus the tumor DNA safety net groups (Fig. 4). The majority of total cases in the traditional genetic counseling group (tumor variant only, or a tumor + germline LP/P variant) were comprised of ovarian, breast, prostate, colorectal, and pancreatic cancers (in decreasing frequency with all of these tumor types exceeding frequencies found in the tumor DNA safety net group), while the total cases in tumor DNA safety net group was comprised mainly of non-small cell lung cancer (NSCLC), “other”, sarcoma, esophagogastric, renal, hepatobiliary, melanoma, and CNS/PNS tumors (in decreasing frequency with all of these tumor types exceeding frequencies found in the traditional genetic counseling group; Fig. 4a). As shown in Fig. 4b, the percent of germline LP/P variants within the traditional genetic counseling and tumor DNA safety net groups was similar for BRCA2 (40% versus 32%), PALB2 (3% versus 8%), and Lynch syndrome genes (collectively 19% versus 13%). Germline LP/P ATM variants comprised a higher proportion of the tumor DNA safety net group compared to the traditional genetic counseling group (42% versus 13% Fig. 4b) while germline LP/P BRCA1 variants comprised a higher percentage of the traditional genetic counseling group compared to the tumor DNA safety net group (33% versus 16%; Fig. 4b).

## Discussion

There is a growing body of literature demonstrating that variants found on tumor DNA sequencing tests for certain high- and moderate-risk hereditary cancer risk genes are also frequently present in the germline. This has been demonstrated in paired germline-tumor tumor DNA analysis from large unselected groups of patients with diverse cancers [3, 4] as well as work focused on specific cancer types [10–14]. Both approaches have demonstrated that tumor variants in certain hereditary cancer genes are likely to be present in the germline across a large spectrum of tumor types. Tumor variants in high-risk genes such as BRCA1/2, PALB2, and Lynch syndrome genes (MLH1, MSH2, MSH2, and PMS2) are germline in a significant fraction of cases among unselected and cancer-specific approaches. Our work shows a similar frequency (approximately 1/3) of tumor variants are present in the germline for these high-risk genes among a broad range of stage III/IV cancers from an ethnically diverse patient population. In addition, we demonstrate a similar frequency of germline variants among patients with tumor variants in the moderate risk gene ATM, which has not been examined extensively in previous studies.

Our work also highlights that tumor variants in hereditary cancer genes have a similar likelihood to be germline for both typical and atypical tumor types for various hereditary cancer risk genes. As expected, BRCA1/2 variants comprised the majority of the tumor/germline variants in patients with stage III/IV breast, ovarian and pancreatic cancers; however, we also detected tumor/germline BRCA2 variants in atypical cancers such as colorectal, esophagogastric, NSCLC, and sarcoma. Most all of these germline variants in atypical cancers were identified through our tumor DNA safety net. Many “high-risk” hereditary cancer risk genes may cause small but statistically significant increases in cancer risks for atypical organs. For example, germline variants in BRCA2 and ATM may cause increased risk for lung cancer [13, 15–17] and sarcoma [18, 19], two tumor types in our study that revealed a significant number of tumor (and germline) variants in hereditary cancer risk genes not typically associated with these tumor types. Although ATM is typically most associated with a moderate increase in female breast cancer risk [20], we found tumor/germline variants in ATM distributed across virtually all tumor types studied. Although our study does not address whether hereditary cancer genes such as BRCA2 or ATM increase lung cancer or sarcoma risk—or whether patients with these hereditary cancer risk genes developed these atypical tumors by chance and/or environmental features—it is clear that the presence of tumor hereditary cancer gene variants in atypical tumors is an opportunity to diagnose hereditary cancer.

The use of a universal tumor DNA screen for hereditary cancer highlights the effectiveness to an extent of typical genetic counseling approaches for the diagnosis of hereditary cancer. In KPSC, we have relied extensively on personal/family history guidelines (e.g. NCCN) to drive patient referrals and determine eligibility for germline testing with a typical rate of LP/P variants among eligible patients between 10 and 15% [21]. Patients with tumor variants and typical hereditary tumor types (e.g. breast, ovarian, pancreatic, prostate, colorectal tumors) were much more likely to have undergone germline testing prior to having a tumor DNA test than were patients who had tumor variants in atypical tumor types (e.g. hepatobiliary, bladder/renal, NSCLC, sarcoma, and “other” tumors). Not surprisingly, patients who had tumor variants and had previously completed germline testing (due to their personal/family history of cancer) demonstrated a very high rate of germline LP/P results (54%). Among patients with a tumor variant in a hereditary cancer gene who had been previously diagnosed with a germline variant, 99% of these patients met NCCN personal and/or family history criteria for testing.

Although personal/family history criteria have provided a useful diagnostic framework for genetic clinicians, it is becoming clear that relying on this approach does not have optimal sensitivity. Patients diagnosed with hereditary cancer through our universal tumor DNA safety net had a higher proportion of atypical cancers (such as lung, sarcoma, and “other” tumors), had a higher frequency of male biological sex, and failed to meet current NCCN personal/family history criteria in roughly 2/3 of cases. Several other studies on paired tumor/germline hereditary cancer testing have also demonstrated that a significant fraction of patients with hereditary cancer ascertained through tumor DNA information failed to be captured by traditional genetic counseling methods and/or had atypical tumor types [10, 12, 22–27]. Our work along with published data suggests that tumor-based screening methods to diagnose hereditary cancer are able to capture a large number of patients missed by personal/family history-based approaches.

One limitation of our study is the relatively large number of patients with tumor variants who did not complete testing. All patients who underwent tumor DNA sequencing had advanced disease, and patient death and/or poor health were the most common causes reported to providers for failure to complete germline testing. Anecdotally, direct outreach to patients by a genetic provider also led to apprehension to complete testing in some cases. Subsequently, we have modified our approach in several ways that has improved testing uptake, such as more rapid patient contact after obtaining tumor results, and increasing patient awareness that their treating oncologist is an active part of the screening program (data not shown).

Several different tumor DNA screening strategies have been presented in the literature. A number of groups have used a paired tumor/germline testing strategy for all patients undergoing tumor testing, which maximizes sensitivity but requires a large amount of germline testing and genetic counseling resources [3, 4]. Other groups have presented a “multidisciplinary tumor board” review of tumor DNA test results [23, 28]; however, this type of approach requires manual review of all tumor test results and significant practitioner resources to staff a molecular tumor board. Our more automated approach does not require a manual review of tumor DNA test results or a multidisciplinary tumor board review- only a computer-based database query by a data analyst for relevant tumor variants in patients who had not previously undergone germline testing was necessary to generate patient lists for genetic counseling outreach. Such an approach optimizes utilization of germline testing and genetic counseling resources. No matter what specific approach is taken, we would argue that universal tumor DNA screening for hereditary cancer should be more broadly utilized within healthcare systems to augment current diagnostic approaches. Guidelines on when to consider germline testing in light of tumor DNA test results have been proposed and will likely evolve as more data in this area emerge [5, 29].

## Electronic Supplementary Material

Below is the link to the electronic supplementary material.

Supplemental Figure 1: Universal tumor DNA safety net protocol. Patients with stage III/IV cancers who underwent tumor DNA testing had test results that were returned to oncologists to help guide treatment. Information about tumor DNA variants were kept in a centralized database. Patients with tumor variants in ATM, BRCA1, BRCA2, MLH1, MSH2, MSH6, PALB2, and/or PMS2 were identified through regular searches of the tumor variant database. Patients who had previous germline testing were not contacted. Patients who had not previously undergone germline testing were contacted by a genetics provider and offered genetic counseling and germline testing.

## Data Availability

The datasets generated during and/or analysed during the current study are available from the corresponding author on reasonable request.
