# Supplementary figures and images for: Next-generation universal hereditary cancer screening: implementation of an automated hereditary cancer screening program for patients with advanced cancer undergoing tumor sequencing in a large HMO
Source: Fam Cancer. 2022 Oct 20;22(2):225–35. doi: 10.1007/s10689-022-00317-w (PMC10020326; doi:10.1007/s10689-022-00317-w)

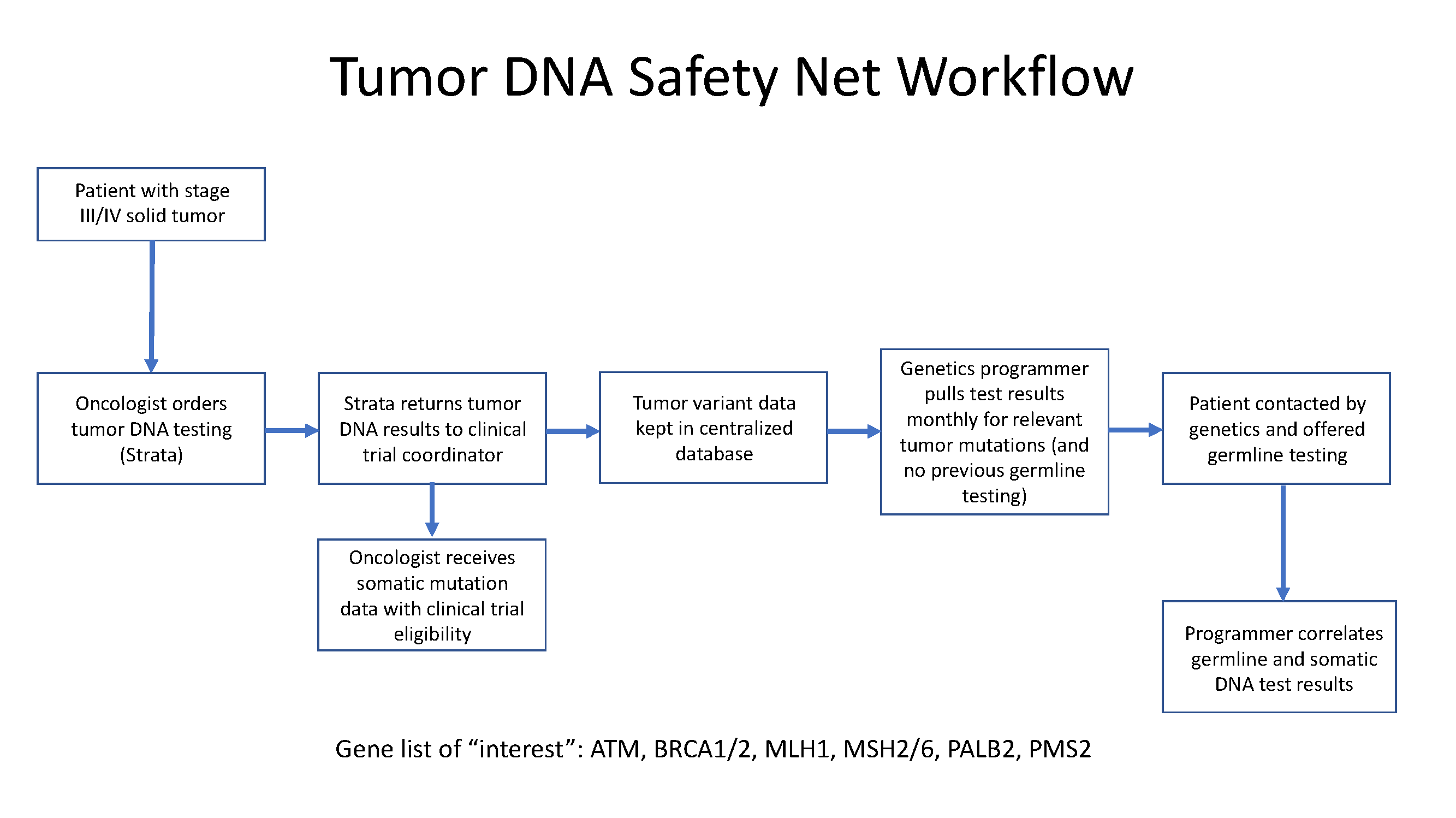

Supplement: Supplementary file 1 — Supplemental Figure 1: Universal tumor DNA safety net protocol. Patients with stage III/IV cancers who underwent tumor DNA testing had test results that were returned to oncologists to help guide treatment. Information about tumor DNA variants were kept in a centralized database. Patients with tumor variants in ATM, BRCA1, BRCA2, MLH1, MSH2, MSH6, PALB2, and/or PMS2 were identified through regular searches of the tumor variant database. Patients who had previous germline testing were not contacted. Patients who had not previously undergone germline testing were contacted by a genetics provider and offered genetic counseling and germline testing. [file 10689_2022_317_MOESM1_ESM.tif]
